# Interruptions and multitasking in anaesthesia nursing: a prospective observational study of cognitive strain and workflow patterns

**DOI:** 10.1136/bmjoq-2025-003972

**Published:** 2026-03-02

**Authors:** Carlos Ramon Hölzing, Paul Heilgenthal, Florian Sellmann, Charlotte Meynhardt, Tobias Grundgeiger, Rainer Scheuchenpflug, Patrick Meybohm, Oliver Happel

**Affiliations:** 1 Department of Anaesthesiology, Intensive Care, Emergency and Pain Medicine, University Hospital Würzburg, Würzburg, Germany; 2 Psychological Ergonomics, University of Würzburg, Würzburg, Germany; 3 Institute of Psychology, University of Würzburg, Würzburg, Germany

**Keywords:** Patient safety, STRESS, Nurses, Communication

## Abstract

**Background:**

In critical fields such as anaesthesiology, maintaining uninterrupted focus during key procedures, particularly during critical phases of anaesthesia care, such as induction and extubation, is crucial for patient safety. Multitasking and interruptions in healthcare settings have been linked to increased error rates and reduced efficiency. This study comprises two parts: (1) an objective observational analysis of multitasking and interruptions and (2) an exploratory examination of their relationship to perceived work-related stress, perceived error risk and job satisfaction.

**Methods:**

In this prospective observational study, 19 anaesthesia nurses at the University Hospital in Würzburg were observed during 30 field sessions. The study used the Work Observation Method by Activity Timing application for real-time recording and classification of tasks into primary activities (core clinical tasks), secondary activities (parallel tasks, ie, multitasking) and interruptions (externally triggered interruptions leading to task cessation). Spearman’s rank correlation coefficients were calculated to examine associations between observational data and subjective ratings.

**Results:**

Interruptions accounted for 4% of the total observation time, with secondary activities being performed during 8.5% of the time. The average duration of interruptions was 36 s. Primary activities constituted 74.36% of all tasks, with communication-related interruptions being the most frequent. Preparatory work comprised more than half of the total duration of primary activities. Communication tasks were the dominant event during secondary activities, with a significant number of steps associated with them. On a subjective level, a strong positive correlation was found between perceived stress and error potential.

**Conclusions:**

Interruptions and secondary activities were common in anaesthesia nursing workflows but accounted for only a small proportion of total working time. Most interruptions involved communication required for perioperative coordination. Step-based movement estimates showed substantial physical workload, with walking activity unevenly distributed across task categories and predominantly occurring during primary activities.

**WHAT IS ALREADY KNOWN ON THIS TOPIC**

**WHAT THIS STUDY ADDS**

- In anaesthesia nursing, interruptions occurred frequently but were brief and constituted only a small proportion of total working time. Most interruptions and multitasking episodes were communication-related, underscoring the central role of coordination in perioperative workflows.
- Step-based movement estimates revealed considerable physical workload and high movement density concentrated in key task categories, offering a novel quantitative view of physical demands in anaesthesia care.

**HOW THIS STUDY MIGHT AFFECT RESEARCH, PRACTICE OR POLICY**

- Implementation of structured communication protocols and systematic equipment-readiness routines may reduce avoidable workflow disruptions, improve efficiency and support staff well-being and patient safety in perioperative nursing environments.

## Background

 Anaesthesia care environments are defined by time-critical procedures, complex team coordination and high cognitive demands. Maintaining an uninterrupted focus during key procedures, particularly during the induction phase of general anaesthesia, is essential for patient safety. The induction process involves a series of precise, ordered steps conducted by medical and non-medical staff, where any deviation or interruption could have serious implications.^1^

Cognitive psychology research extensively documents how multitasking negatively influences performance, with the degree of impact contingent on the similarity of the tasks, their demand levels and the individual’s familiarity with the tasks.^2^,^4^ These effects are notably pronounced within clinical settings, where the propensity for multitasking significantly elevates the risk of errors.^5 6^ Further, Li *et al* and Westbrook *et al* identified a positive link between interruptions and the incidence of errors, emphasising the inherent risk associated with these interruptions.^7 8^ Similarly, Grundgeiger *et al* demonstrated in a simulation study that interruptions during critical phases of anaesthesia can disrupt task execution and lead to safety-compromising omissions.^9 10^ Investigations into multitasking in healthcare reveal that introducing even seemingly simple concurrent tasks can markedly reduce work efficiency and accuracy.^8 11^

Anaesthesia nursing involves a complex blend of patient care, technical preparation and continuous coordination under conditions of rising surgical volumes and persistent staffing challenges. This surge places significant pressure on anaesthesia care nurses, who not only manage direct patient care (eg, assistance during medical procedures, vital checks, postanaesthesia care unit (PACU) support) but also prepare the anaesthesia workplace and handle organisational tasks like procuring and maintaining medical devices, restocking consumables and ensuring thorough documentation.^12^ Their broad scope of duties requires vigilance, adaptability and effective communication. In contrast to nurse anaesthetists in countries like the USA, anaesthesia care nurses in Germany do not provide independent anaesthesia care. Instead, they support a physician anaesthesiologist throughout the perioperative process, including preparation, induction and patient monitoring.

Although research into the full extent of workflow interruptions in medical contexts is not yet comprehensive, existing studies suggest significant implications. Weigl *et al* found a correlation between the frequency of interruptions and increased workload reports among physicians, indicating that such disruptions may be a considerable source of occupational strain in healthcare settings.^13^ At the same time, frequent or simultaneous tasks, sometimes occurring every 1–2 min, can multiply the risk of stress and potential errors, especially under the unpredictable conditions that are inherent in the operating room (OR) environment.^14^

Despite these findings, much of the literature on interruptions and multitasking in the OR has focused on physicians or entire surgical teams, with fewer studies exclusively targeting anaesthesia care nurses.^15^ In some healthcare systems, nurse anaesthetists operate semiautonomously, performing comprehensive anaesthesia care, whereas in other contexts, including many European countries, anaesthesia care nurses mainly assist a physician anaesthetist. This divergence in role definitions likely translates into different patterns and frequencies of both interruptions and multitasking.

A major concern is that even brief workflow disruptions can increase error risk and strain on cognitive capacity.^11^ Li *et al* highlighted the adverse effects on task completion time, work strategies and decision-making processes, pointing to the broad and detrimental impact of interruptions on clinical operations.^7^ Given that higher stress levels positively correlate with error likelihood, understanding the nature and frequency of interruptions in anaesthesia nursing is of paramount importance.^13^

In addition to cognitive demands, anaesthesia nursing involves a substantial physical workload that is shaped by frequent room transitions, equipment retrieval and coordination across distributed perioperative spaces. Movement patterns, therefore, represent a complementary workload dimension that can be quantified using step-based estimates derived from observed location changes. Communication is another core workflow element in this setting because coordination relies on frequent brief exchanges that can occur as interruptions or parallel activities. This study comprises two parts. First, we provide an observational description of anaesthesia nursing work, including task distributions across primary activities, secondary activities and interruptions, their subcategories and step-based movement estimates derived from recorded location transitions. Second, we explore the associations between observed workflow indicators and self-reported work-related stress, perceived error potential and job satisfaction.

## Methods

### Study design

This prospective study combined a direct observational time and motion design with a short provider survey. The observational component recorded the workflow of anaesthesia nurses (ANs), while the survey assessed subjective perceptions related to the observed work period and its contextual conditions. Each observation session represented a single observation unit and involved the continuous monitoring of one AN during a routine clinical work shift. The observational component included a predefined task taxonomy with subcategories and an additional step-based movement estimate derived from transitions between recorded locations. The paper was written using the Strengthening the Reporting of Observational Studies in Epidemiology checklist (online supplemental file 1).^16^ A clinical trial number was not applicable.

### Setting

Data were collected from 10 August to 2 November 2021 at the central OR of the university hospital (tertiary care centre) in Würzburg, Germany. The observations were made using the Work Observation Method by Activity Timing (WOMBAT) in a section of the OR, covering scheduled surgeries in trauma, urology and general surgery.^17^,^19^ This section includes six ORs, corresponding induction rooms, storage, laboratories, offices, a PACU, gowning areas and a break room. The study period included regular weekday working hours (7:00–16:00).

### Observation procedure and data recording

Each session captured the full range of tasks performed by ANs during that period, including patient preparation, equipment setup, intraoperative support (eg, assistance during induction/intubation) and postprocedural documentation or handovers, depending on the timing within the workflow. Observations were conducted by two trained independent observers (ie, members of the study team with no clinical responsibilities during the session), all of whom were instructed in the use of the WOMBAT method and familiarised with the anaesthesia workflow through pilot sessions prior to data collection. The WOMBAT is a structured, real-time observational tool designed to quantify clinical work processes through timestamped activity logging across multiple dimensions.^17^,^19^ It has been validated and applied in various healthcare settings to assess workload, interruptions, multitasking and communication patterns.^20^ In our study, we used the WOMBAT iPad application (V.3, release date: March 2021) to record observed anaesthesia nursing tasks across four predefined dimensions: (1) task category (primary or secondary activities), (2) location, (3) interruption type (if applicable) and (4) optional comments. Task duration was automatically logged by the application. Location logging also enabled a step-based movement estimate by translating successive room transitions into predefined step equivalents measured on site before data collection. Observers selected each activity in real time from a predefined coding taxonomy adapted from Weigl *et al* and tailored to anaesthesia nursing through pilot observations.^14^

### Participants

The study participants were ANs working in the relevant section of the OR during the data collection period. The ANs were selected using a convenience sample. Patients and other staff were not observed, but they were informed about the observations and given the opportunity to refuse data collection. Inclusion criteria were a completed professional nursing qualification, assignment to a regular day shift, age over 18 years and written informed consent.

### Variables

The study design comprised two components. First, an observational analysis describing workflow patterns based on direct task measurement, including distributions of task subcategories and step-based movement estimates derived from location transitions. Second, an exploratory correlational analysis based on a short study-specific questionnaire, assessing subjective ratings collected before and after each observation session. Pre-session ratings captured contextual baseline factors that could shape the subsequent work period, namely fatigue, perceived personnel adequacy and non-occupational stress. Postsession ratings referred explicitly to the observed work period and, therefore, assessed work-related stress, perceived error potential and job satisfaction immediately after the session. All recorded tasks were categorised into three distinct types: (1) primary activities, (2) secondary activities (i.e., multitasking) and (3) interruptions. The categorisation was based on a validated observation tool developed by Weigl *et al* and adapted to the specific context of anaesthesia nursing.^13 14^ To ensure contextual appropriateness, activity and room categories were refined through exploratory pilot observations. Based on these sessions, all categories and subcategories were standardised and programmed into the WOMBAT application. The classification of an event as an interruption was based on the validated definition by Weigl *et al*, requiring an unplanned, externally triggered event that leads to a visible cessation of the ongoing task for at least two seconds.^13^ Secondary activities (multitasking) were defined as distinct parallel tasks performed without halting or abandoning the ongoing primary activity. For example, speaking to another staff member about future procedures while simultaneously preparing an intravenous line was classified as a secondary activity. Movement data were estimated by translating transitions between predefined locations into approximate step equivalents, based on the room layout and standard gait metrics. In addition, self-reported variables were collected using a structured paper-based questionnaire administered before and after each observation session. These variables included subjective work-related stress level (post-observation), perceived error potential, job satisfaction, tiredness, perceived sufficiency of personnel and presence of non-occupational stress, as well as demographic information such as age, gender, years of work experience and whether the participant held a shift management role. All self-assessments were rated on an 11-point Likert scale ranging from 0 (‘not at all’) to 10 (‘very strong’).

### Data sources and measurement

Inter-rater agreement was assessed for the categorisation of primary activities and interruptions using Cohen’s kappa. The resulting agreement coefficient was κ=0.789, indicating substantial agreement between observers.^20^ Participants completed a short questionnaire immediately before and again immediately after each observation session. Each questionnaire consisted of three single-item ratings on a 0–10 Likert scale. The pre-observational questionnaire assessed subjective fatigue, perceived personnel adequacy and non-occupational stress. The post-observational questionnaire assessed perceived error potential, job satisfaction and work-related stress. The latter two items specifically referred to the preceding 90 min work period. Job satisfaction was defined in terms of personal goals and expectations related to the observed tasks. Additionally, demographic information such as age, gender, years of professional experience and shift leadership was collected. The questionnaire was iteratively developed by the project team to ensure clarity and contextual appropriateness, but it was not pilot-tested. The questionnaire used for these ratings and demographic data was developed specifically for this study and is provided as online supplemental file 2.

Primary activities, secondary activities and interruptions were recorded using the WOMBAT,^19^ application on a tablet computer (Apple iPad). Within WOMBAT, each task was categorised along four dimensions: location, activity type (primary or secondary activities), interruption category and optional notes. The duration of each activity was automatically logged by the software.

Step count was estimated using an automated approximation method based on location data recorded in WOMBAT. Each task was assigned to a specific room, and predefined step counts between adjacent rooms, measured by the research team prior to data collection, were used to approximate inter-room walking distances. Sequential room transitions extracted from the WOMBAT dataset were used to retrospectively calculate total step counts per session.

At the session level, the total number of primary, secondary and interrupted activities, as well as interactions with colleagues, physicians, the OR team and patients, was computed. These were converted into hourly rates to allow comparisons between observation sessions of different lengths. These normalised indicators, expressed as rates per hour to account for varying observation durations, formed the basis for further statistical analyses. To enable linkage between observational data and questionnaire responses, each session was assigned a unique numeric pseudonym.

### Bias

During the data collection period, the limited availability of different individual staff members led to repeated observations of some ANs. Six participants were observed two times, one was observed three times and one was observed four times. The presence of observers may have altered the behaviour of staff members.^21^ As multiple sessions were conducted with some individuals, each observation session was treated as an independent unit of analysis reflecting the situational workflow conditions of that session rather than stable individual behaviour. We did not aim to analyse within-person behavioural changes across repeated observations, as the study was designed to characterise workflow patterns at the session level rather than individual traits. Consequently, derived workload subcategories were interpreted as context-specific patterns and not as individual-level characteristics.

### Study size

As this was an exploratory observational study, no formal a priori sample size calculation was conducted. The sample size of n=30 observation units is based on similar observational studies. Observation sessions were scheduled to last 90 min to avoid observer fatigue in a dynamic environment.^22^,^24^ The observation times were determined based on staffing levels at various times and covered the period from 7:00 to 16:00 on weekdays.

### Statistical methods

All statistical analyses were performed using IBM SPSS Statistics V.27. Descriptive statistics were used to summarise the frequency and duration of primary activities, secondary activities and interruptions. In addition, we report two-sided 99% CIs for interruptions and secondary activities at the observation-unit level (see online supplemental table S4). These values reflect clustering by nurse. As only one observation was available for most nurses (ie, sessions were not systematically nested within nurses), no formal random effects model could be applied. Instead, CIs were derived from session-level distributions, supplemented by minimum and maximum values to reflect the full range of observed variability. Results are reported as mean±SD. Spearman’s rank correlation coefficients were calculated to examine the associations between observational data and subjective ratings. To account for multiple testing in the correlation analyses, p values were adjusted using the Bonferroni-Holm procedure.

## Results

A total of 30 observation sessions were conducted, each accompanied by a corresponding provider survey completed before and after the observation. The average duration per observation unit was 1:34:46±0:08:58 hours. A mean of 60.97±17.55 activities per observation unit was recorded. A cumulative total of 47 hours and 23 min of observation time was recorded across 30 sessions (see table 1). 19 participants were recruited from the nursing staff of the department, of whom 12 were female and 7 male. The mean age of the participants was 37.47±10.44 years, and their work experience accounted for 8.40±7.76 years. 8 participants were certified general nurses with anaesthesia experience, and 11 had completed additional specialised training in anaesthesia and intensive care or held a degree as anaesthesia technical assistants.

**Table 1:** Descriptive overview of activities and interruptions

	Primary activities	Interruptions	Secondary activities	Total
Duration				
Hours	45:36:13	1:46:55	4:25:01	51:48:09
Proportion (%)	88.03	3.44	8.59	100.00
average duration per task (sec)	115	36	54	115
Frequency				
n (task episodes)	1430	177	292	1829
Proportion (%)	74.36	9.67	15.97	100.00
Task rate per hour	28.70	3.74	6.16	38.60
Steps walked				
n (steps)	23 326	692	283	24 301
Proportion (%)	95.99	2.85	1.16	100.00
Rate per hour	511.50	388.34	79.01	514.23

### Descriptive data

Primary activities accounted for 74.36% (n=1430) of all recorded tasks, with an average duration of 115 s. Interruptions represented 9.67% (n=177) of tasks and had an average duration of 36 s. Secondary activities made up 15.97% (n=292) of all tasks, with an average duration of 54 s. In terms of time proportion, primary activities constituted 88.03% of the total observation time, interruptions 3.44% and secondary activities 8.53% (see table 1). The mean activity rate was 28.70 primary activities per hour, 3.93 interruptions per hour (99% CI 2.42 to 5.43) and 6.14 secondary activities per hour (99% CI 3.74 to 8.54). These CIs illustrate the variability across sessions (see online supplemental table S4). A total of 24 301 steps were documented. Of these, 95.99% were associated with primary activities, 2.85% with interruptions and 1.16% with secondary activities. Steps were directly assigned to the respective activity categories based on observed room transitions and predefined step counts measured prior to data collection. To allow comparison across task categories with different time proportions, activity-specific step counts were normalised to rates per hour of activity duration. This corresponds to an average of 517.90 steps per hour of primary activity time, 388.34 steps per hour of interruption time and 79.01 steps per hour of secondary activity time, with a total average of 514.23 steps per hour across the entire observation period.

**Figure 1:**
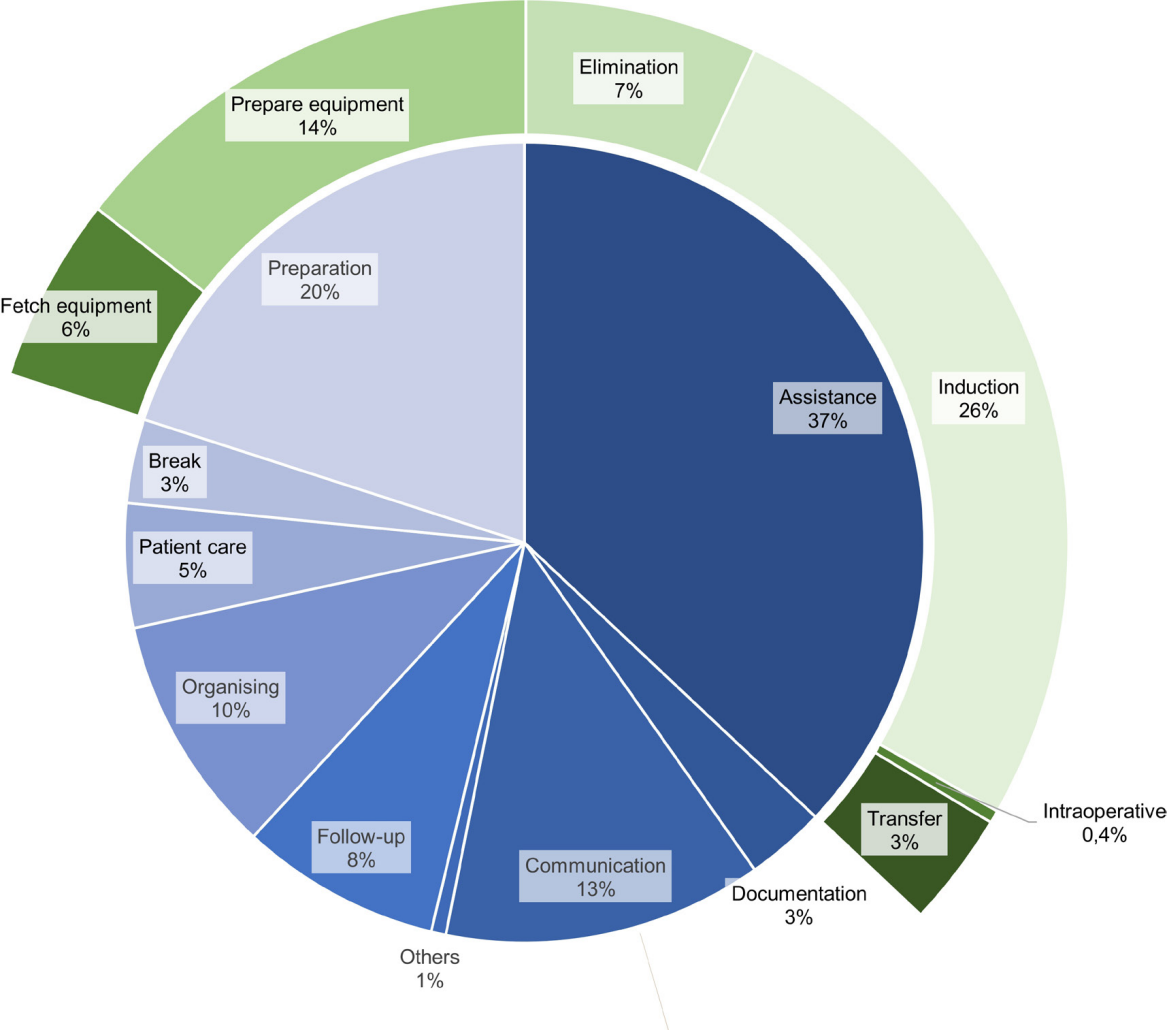
Relative distribution of subcategories by total duration. The inner ring shows the five main categories (eg, direct patient care, documentation and coordination), while the outer ring represents the corresponding subcategories within each main category.

Figure 1 shows the relative distribution of basic activity subcategories by total duration. The categories ‘assistance activities’ and ‘preparatory work’ accounted for the largest proportions. Subcategories of these two groups were included for further detail. A detailed overview of all subcategories is provided in online supplemental file 3.

**Figure 2:**
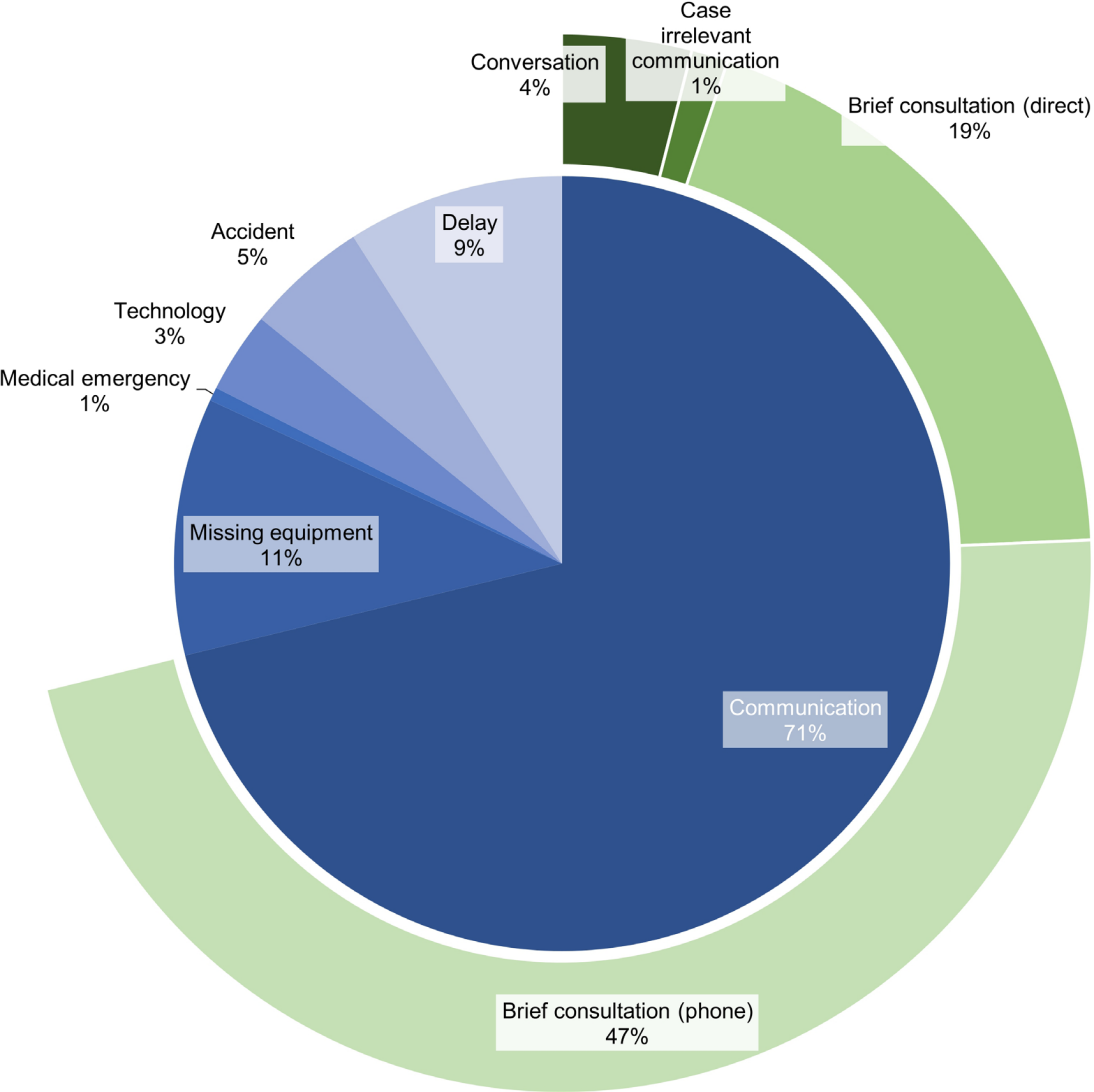
Frequency distribution of interruption subcategories. The inner ring shows the main subcategories of secondary activities (eg, verbal communication and equipment management), while the outer ring contains further detail for selected categories.

Figure 2 illustrates the frequency distribution of interruption subcategories. Due to their generally brief duration, interruptions were analysed by count rather than time. Communication-related interruptions were most common, primarily consisting of telephone-based and face-to-face interactions.

**Figure 3:**
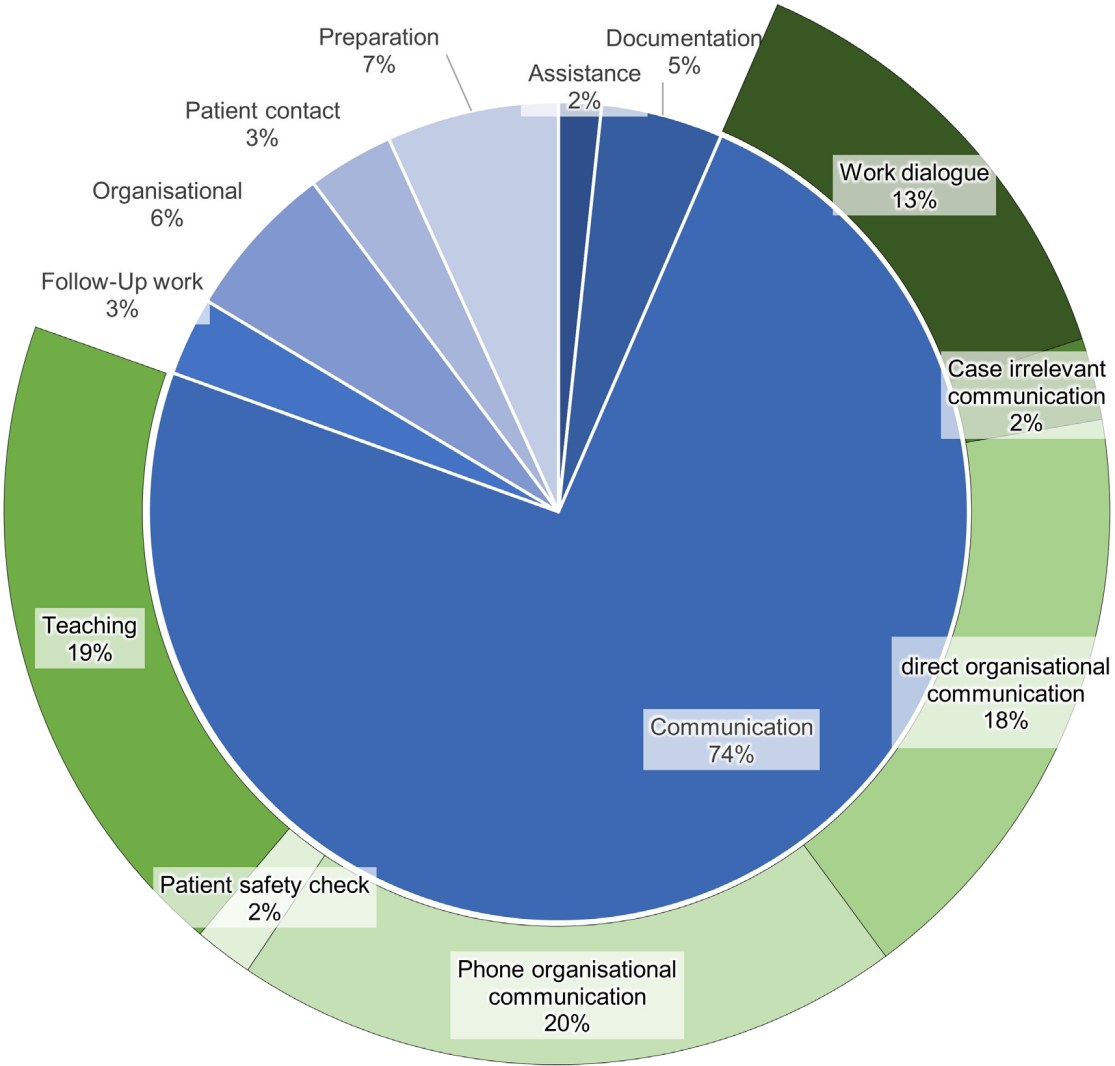
Frequency of secondary activities by category. The inner ring distinguishes the main sources of interruptions (eg, physician requests, patient queries and alarms), and the outer ring shows more granular causes or subcategories of interruptions.

Figure 3 presents the frequency of secondary activities by category. Communication tasks also predominated in this category, with further breakdowns into subcategories shown.

### Exploratory analysis of relevant variables

Correlation analyses were conducted to examine the relationships between variables derived from the 30 observation sessions and subjective evaluations obtained via postsession questionnaires, as detailed in online supplemental tables S5 and S6 in online supplemental file 3. The variables included both contextual factors (eg, shift characteristics and professional experience) and subjective ratings. The variables were collected via short pre-observation and post-observation questionnaires. Before each session, ANs rated their non-occupational stress, current fatigue and perceived personnel adequacy. After each session, they provided ratings of job satisfaction, perceived error potential and work-related stress level.

## Discussion

This study provides a detailed analysis of the distribution of primary and secondary activities, as well as interruptions, in the daily routines of anaesthesia care nurses in a busy university hospital’s OR. Our step-based analysis adds a descriptive physical workload perspective to the understanding of anaesthesia nursing workflows.

### Workload distribution and communication patterns

Because perioperative work relies on continuous coordination, we expected communication to appear prominently both as a source of interruptions and as parallel activity within multitasking episodes. With an average of 514.23 steps per hour during observed work periods, equivalent to over 4100 steps per 8-hour shift, ANs demonstrated a substantial amount of walking activity during routine perioperative work. By linking step-based movement estimates with task categories, our analysis provides novel insights into physical workload distribution, demonstrating how walking activity is unevenly distributed across primary activities, interruptions and secondary activities rather than occurring uniformly throughout the workflow. While primary activities accounted for 511.50 steps/hour (95.99% of total steps), interruptions showed disproportionately high step density at 388.34 steps/hour despite constituting only 3.44% of observation time (see online supplemental table S3). The relatively high step density during interruptions reflects that some interruptions occurred during transit or initiated immediate movement, most often related to coordination or equipment retrieval. Among primary activities, the most time-consuming and frequent subcategories were assistance during anaesthesia (eg, airway or intravenous access preparation), preparatory work (eg, retrieving and setting up equipment) and follow-up activities (eg, cleaning and documentation). These three categories alone accounted for over 60% of the total basic activity time. Conversely, secondary activities presented markedly reduced mobility at 79.01 steps/hour, indicating stationary task execution during parallel processing. The total number of tasks recorded across the 30 sessions was 1829, with primary activities comprising approximately three-quarters of these tasks. Communication tasks emerged as the dominant component among both interruptions and secondary activities. This is consistent with previous studies emphasising the centrality of communication in perioperative environments.^23 25 26^ Furthermore, communication tasks are the most frequent and time-consuming secondary activity events (74.5%), with a remarkable mean number of steps (n=283) associated with them. This extends prior work by quantifying the physical toll of coordination efforts.^27^ Furthermore, prior work suggests that multitasking in healthcare is constrained by task characteristics and modality, as described by Douglas *et al*.^28^ Our data remain descriptive and do not allow conclusions regarding cognitive load or performance effects. Secondary activities organising tasks represented 5.9% of the total time with a considerable mean step count (36 steps). This finding echoes the work of Currie and Carr Hill, who discussed the complexities inherent in the organisational tasks of nurses and their impact on clinical efficiency.^29^

### Equipment-related disruptions and implications for safety

The near equivalence in interruptions per hour with results from the literature (our study: 3.73, Olin *et al*^23^: 3.7) indicates a potentially universal challenge within anaesthesia care environments. Interruptions in nursing care, particularly during medication administration, are a critical aspect that can significantly impact patient safety and healthcare delivery efficiency.^30^ Interruptions were not evenly distributed across tasks but predominantly communication-related and clustered around coordination and preparatory activities. This pattern is consistent with recent observational evidence showing that interruption likelihood varies by task type rather than occurring randomly. Marsall *et al* demonstrated task-dependent interruption patterns in acute care, particularly during documentation and coordination activities.^31^ Furthermore, the predominance of communication-related interruptions in our study aligns with findings by Weigl *et al*, who highlighted the complexity and critical nature of communication in high-stakes healthcare environments like the OR.^13^ Filer *et al* have shown that the establishment of no-interruption zones (NIZs) reduces interruptions and allows activities to be completed in sequence.^32^ In contrast, the interruption rate in high-acuity settings like Intensive Care Units exceeds 14/hour.^33 34^ This discrepancy may reflect role-specific task structures in anaesthesia nursing, in which highly standardised procedural sequences leave fewer opportunities for ad hoc task interruption during certain phases of care. It is also important to acknowledge that not all interruptions are inherently detrimental. Certain safety checks, such as the surgical timeout, represent intentional workflow interruptions that are central to patient safety. Similarly, intraoperative priority shifts, such as a request to reposition the patient during acute bleeding, may temporarily disrupt medication preparation but are critical to surgical and patient outcomes. In this sense, what counts as an interruption is context-dependent. In our observations, patient safety checks were explicitly coded as a separate category (see online supplemental tables S1 and S2), confirming their presence as structured, purposeful tasks rather than disruptive events. To reduce the impact of interruptions, several mitigation strategies have been proposed and can be adapted to the context of anaesthesia nursing. These include establishing NIZ during critical phases such as induction, improving access to necessary equipment and implementing structured communication protocols like SBAR (structured communication scheme) to reduce unnecessary queries.^35^ Equipment-related interruptions, characterised by their extended duration and high step count, suggest significant inefficiencies in equipment management, a concern similarly identified by Catchpole *et al* as a major disruptor in surgical workflows.^36^ Equipment-related interruptions were observed but constituted only a limited proportion of the overall interruptions. These findings are reported descriptively and should not be interpreted as evidence for specific workflow deficiencies or intervention targets.^37^ Garrouste-Orgeas *et al* advocate for interventions aimed at reducing work-related stress, reinforcing the idea that addressing stress is crucial for patient safety and healthcare quality.^38^ Importantly, the high proportion of communication-related interruptions reflects essential coordination processes in perioperative care rather than avoidable workflow deficiencies.

### Implications for future research and clinical practice

These findings suggest several directions for future research. Observational workflow data may be used to examine task-specific interruption patterns and transitions across perioperative phases. Larger and multicentre studies could allow modelling of task dependencies and contextual moderators of interruptions and multitasking. Combining task-based observations with phase-sensitive risk markers may further clarify when workflow disruptions are potentially safety relevant. For clinical practice, the predominance of communication-related interruptions and secondary activities indicates that optimisation of coordination structures is more relevant than general interruption reduction. For clinical practice, the findings characterise communication and coordination as core elements of routine anaesthesia nursing work. Interruptions and parallel activities were observed in relation to task context and workflow phase, reflecting routine workflow organisation rather than discrete targets for reduction.

### Limitations

The sample size of 30 observation sessions and repeated observations of individual participants can be considered modest compared with larger multicentre observational studies. While sufficient for exploratory analyses, this may limit the statistical power for detecting smaller effects and constrain the generalisability of findings. Generalisability is constrained due to the single-centre design. Although each session was conceptualised as a situational workload snapshot, the possibility of intra-individual behavioural patterns influencing the results cannot be excluded. Furthermore, the subjective ratings relied on single-item measures. Our descriptive analysis did not account for clustering at the nurse level. We did not perform a formal random-effects analysis, as the study was not powered for multi-level modelling, and repeated observations were available for only 8 of 19 nurses and were limited in number (see the Section Bias). A substantially larger number of observations would be desirable in future studies to enable more granular analyses across task types, workflow phases or institutional contexts and to increase external validity. Future studies across multiple institutions are recommended to validate and extend these findings. Subjective ratings of stress, error potential and job satisfaction may have been influenced by social desirability, particularly due to the presence of observers during questionnaire completion. The presence of observers may have introduced observation bias (Hawthorne effect). Moreover, interruptions are not always perceived as disruptive; prior studies suggest that nurses may consider some interruptions neutral or even beneficial.^39 40^

## Conclusions

This observational study provides a detailed characterisation of anaesthesia nursing workflows. While interruptions were frequent, they accounted for only a small proportion of total working time and predominantly reflected communication required for perioperative coordination. By integrating task-based observation with subjective ratings, this study provides a detailed characterisation of anaesthesia nursing workflows. Interruptions were frequent but accounted for only a small proportion of total working time and predominantly reflected communication required for perioperative coordination. The findings highlight the central role of task context and communication in shaping routine anaesthesia nursing work.

## Supplementary material

10.1136/bmjoq-2025-003972 online supplemental file 1

10.1136/bmjoq-2025-003972 online supplemental file 2

10.1136/bmjoq-2025-003972 online supplemental file 3

## Data Availability

Data are available upon reasonable request.

## References

[1] Weinger MB, Slagle JM, Kim RS (2001). A Task Analysis of the First Weeks of Training of Novice Anesthesiologists. *Proceedings of the Human Factors and Ergonomics Society Annual Meeting*.

[2] Allport DA, Antonis B, Reynolds P (1972). On the division of attention: a disproof of the single channel hypothesis. Q J Exp Psychol.

[3] Kahneman D (1973). Attention and effort: citeseer.

[4] Spelke E, Hirst W, Neisser U (1976). Skills of divided attention. Cognition.

[5] Hakimzada AF, Green RA, Sayan OR (2008). The nature and occurrence of registration errors in the emergency department. Int J Med Inform.

[6] Laxmisan A, Hakimzada F, Sayan OR (2007). The multitasking clinician: decision-making and cognitive demand during and after team handoffs in emergency care. Int J Med Inform.

[7] Li SYW, Magrabi F, Coiera E (2012). A systematic review of the psychological literature on interruption and its patient safety implications. J Am Med Inform Assoc.

[8] Westbrook JI, Raban MZ, Walter SR (2018). Task errors by emergency physicians are associated with interruptions, multitasking, fatigue and working memory capacity: a prospective, direct observation study. BMJ Qual Saf.

[9] Grundgeiger T, Liu D, Sanderson PM (2008). Effects of Interruptions on Prospective Memory Performance in Anesthesiology. *Proceedings of the Human Factors and Ergonomics Society Annual Meeting*.

[10] Grundgeiger T, Sanderson PM, Orihuela CB (2010). Distractions and Interruptions in the Intensive Care Unit: A Field Observation and a Simulator Experiment. *Proceedings of the Human Factors and Ergonomics Society Annual Meeting*.

[11] Enz S, Hall ACG, Williams KK (2021). The Myth of Multitasking and What It Means for Future Pharmacists. Am J Pharm Educ.

[12] Heng LMT, Rajasegeran DD, Lim SH (2024). Evaluation of nurse-reported missed care in a post-anesthesia care unit: A mixed-methods study. J Nurs Scholarsh.

[13] Weigl M, Müller A, Vincent C (2012). The association of workflow interruptions and hospital doctors’ workload: a prospective observational study. BMJ Qual Saf.

[14] Weigl M, Müller A, Zupanc A (2011). Hospital doctors’ workflow interruptions and activities: an observation study. BMJ Qual Saf.

[15] Göras C, Olin K, Unbeck M (2019). Tasks, multitasking and interruptions among the surgical team in an operating room: a prospective observational study. BMJ Open.

[16] Elm E von, Altman DG, Egger M (2007). Strengthening the reporting of observational studies in epidemiology (STROBE) statement: guidelines for reporting observational studies. BMJ.

[17] Westbrook JI, Ampt A, Williamson M (2007). Methods for measuring the impact of health information technologies on clinicians’ patterns of work and communication. Stud Health Technol Inform.

[18] Westbrook JI, Creswick NJ, Duffield C (2012). Changes in nurses’ work associated with computerised information systems: Opportunities for international comparative studies using the revised Work Observation Method By Activity Timing (WOMBAT). Ni.

[19] Westbrook JI, Ampt A (2009). Design, application and testing of the Work Observation Method by Activity Timing (WOMBAT) to measure clinicians’ patterns of work and communication. Int J Med Inform.

[20] Walter S, Li L, Westbrook J (2020). A Guide to the Analysis of Work Observation Method by Activity Timing (WOMBAT) Data.

[21] Adair JG (1984). The Hawthorne effect: A reconsideration of the methodological artifact. Journal of Applied Psychology.

[22] Weigl M, Catchpole K, Wehler M (2020). Workflow disruptions and provider situation awareness in acute care: An observational study with emergency department physicians and nurses. Appl Ergon.

[23] Olin K, Göras C, Nilsson U (2022). Mapping registered nurse anaesthetists’ intraoperative work: tasks, multitasking, interruptions and their causes, and interactions: a prospective observational study. BMJ Open.

[24] Antoniadis S, Passauer-Baierl S, Baschnegger H (2014). Identification and interference of intraoperative distractions and interruptions in operating rooms. J Surg Res.

[25] Manojlovich M, DeCicco B (2007). Healthy work environments, nurse-physician communication, and patients’ outcomes. Am J Crit Care.

[26] Miranda M da S, Nascimento FAA do, Lima VN de O (2023). Communication and safe and effective nursing care in surgical center and intensive care: integrative review. *Rev Cienc Saude*.

[27] Thompson BJ (2021). Fatigue and the Female Nurse: A Narrative Review of the Current State of Research and Future Directions. Women’s Health Reports.

[28] Douglas HE, Raban MZ, Walter SR (2017). Improving our understanding of multi-tasking in healthcare: Drawing together the cognitive psychology and healthcare literature. Appl Ergon.

[29] Currie EJ, Carr Hill RA (2012). What are the reasons for high turnover in nursing? A discussion of presumed causal factors and remedies. Int J Nurs Stud.

[30] Johnson M, Sanchez P, Langdon R (2017). The impact of interruptions on medication errors in hospitals: an observational study of nurses. J Nurs Manag.

[31] Marsall M, Schneider A, Wehler M (2025). Associations of professional activities and workflow interruptions in acute care: An observational study in emergency department work. Journal of Patient Safety and Risk Management.

[32] Filer HM, Beringuel BL, Frato KM (2017). Interruptions in Preanesthesia Nursing Workflow: A Pilot Study of Pediatric Patient Safety. J Perianesth Nurs.

[33] Alvarez G, Coiera E (2005). Interruptive communication patterns in the intensive care unit ward round. Int J Med Inform.

[34] Grundgeiger T, Sanderson P, MacDougall HG (2010). Interruption management in the intensive care unit: Predicting resumption times and assessing distributed support. J Exp Psychol Appl.

[35] Raymond M, Harrison MC (2014). The structured communication tool SBAR (Situation, Background, Assessment and Recommendation) improves communication in neonatology. S Afr Med J.

[36] Catchpole KR, Giddings AEB, Wilkinson M (2007). Improving patient safety by identifying latent failures in successful operations. Surgery.

[37] Carthey J, de Leval MR, Reason JT (2001). The human factor in cardiac surgery: errors and near misses in a high technology medical domain. Ann Thorac Surg.

[38] Garrouste-Orgeas M, Perrin M, Soufir L (2015). The Iatroref study: medical errors are associated with symptoms of depression in ICU staff but not burnout or safety culture. Intensive Care Med.

[39] Pape TM, Dingman SK (2011). Interruptions and Distractions During Anesthesia Induction. Plast Surg Nurs.

[40] Sasangohar F, Donmez B, Trbovich P (2012). Not All Interruptions are Created Equal: Positive Interruptions in Healthcare. *Proceedings of the Human Factors and Ergonomics Society Annual Meeting*.

