# Supplementary material for: Interruptions and multitasking in anaesthesia nursing: a prospective observational study of cognitive strain and workflow patterns
Source: BMJ Open Qual. 2026 Mar 2;15(1):e003972. doi: 10.1136/bmjoq-2025-003972 (PMC12959079; doi:10.1136/bmjoq-2025-003972)
Supplement: online supplemental file 2 [file bmjoq-15-1-s002.pdf]

Questionnaire

A1 Biographical

1.1 How old are you? __________ years

1.2 Gender:
☐ Male ☐ Female ☐ Diverse

1.3 What is your highest professional qualification? Qualification: ______________

1.4 Work experience in anaesthesia nursing since graduation: ______ years

1.5 Special role during observation session (e.g., shift leader):
☐Yes ☐ No

A2 Before the Observation

2.1 Personal Situation:
Are you currently impaired in your work due to non-work-related stress (e.g., psychological or social problems, worries, fears)? (Not at all = 0; Absolutely = 10)

0	1	2	3	4	5	6	7	8	9	10
☐	☐	☐	☐	☐	☐	☐	☐	☐	☐	☐

2.2 Fatigue:
How tired and sleepy do you feel at the moment? (Not at all = 0; Absolutely = 10)

0	1	2	3	4	5	6	7	8	9	10
☐	☐	☐	☐	☐	☐	☐	☐	☐	☐	☐

2.3 Staffing Levels:
Would you consider today's staffing levels in your current anaesthesia nursing work area adequate in terms of number and qualifications? (Not at all = 0; Absolutely = 10)

0	1	2	3	4	5	6	7	8	9	10
☐	☐	☐	☐	☐	☐	☐	☐	☐	☐	☐

A3 After the Observation

3.1 Job Satisfaction:
To what extent were you able to achieve your personal goals and expectations in your work during the past 90 minutes? (Not at all = 0; Absolutely = 10)

0	1	2	3	4	5	6	7	8	9	10
☐	☐	☐	☐	☐	☐	☐	☐	☐	☐	☐

3.2 Error Potential Due to Interruptions:
How would you assess the impact of experienced interruptions and multitasking on the occurrence of errors (0 = not at all, conditionally or rather error-inducing; 10 = absolutely a definite cause of errors)?

0	1	2	3	4	5	6	7	8	9	10
☐	☐	☐	☐	☐	☐	☐	☐	☐	☐	☐

3.3 Subjective Stress Level:
To what extent did you feel physically and mentally stressed during the past 90 minutes due to interruptions and multitasking (e.g., phone calls, last-minute changes, disturbances by colleagues, etc.)? (Not at all = 0; Absolutely = 10)

0	1	2	3	4	5	6	7	8	9	10
☐	☐	☐	☐	☐	☐	☐	☐	☐	☐	☐
