# Supplementary material for: Interruptions and multitasking in anaesthesia nursing: a prospective observational study of cognitive strain and workflow patterns
Source: BMJ Open Qual. 2026 Mar 2;15(1):e003972. doi: 10.1136/bmjoq-2025-003972 (PMC12959079; doi:10.1136/bmjoq-2025-003972)
Supplement: online supplemental file 3 [file bmjoq-15-1-s003.pdf]

Table S1 Primary activities

	n	Ø Mean Duration	Σ Total Duration	Ø Mean Steps	Σ Total Steps	Proportion of Total Count	Proportion of Total Duration
Assistance	*273*	*0:03:42*	*16:53:21*	*13.91*	*3755*	*19.10%*	*37.00%*
Emergence	60	0:03:09	3:09:40	14.93	896	4.20%	6.93%
Induction	151	0:04:46	12:00:09	8.96	1353	10.56%	26.32%
Intraoperativ	8	0:01:17	0:10:16	39.00	312	0.56%	0.38%
Transfer	54	0:01:43	1:33:14	22.11	1194	3.78%	3.41%
Documentation	*82*	*0:01:04*	*1:28:02*	*9.35*	*739*	*5.70%*	*3.20%*
Patientrecord	28	0:00:49	0:22:52	5.36	150	1.96%	0.84%
QM	54	0:01:12	1:05:10	10.91	589	3.78%	2.38%
Communication	*333*	*0:01:03*	*5:53:54*	*18.25*	*5657*	*23.30%*	*12.90%*
Work dialogue	97	0:01:28	2:22:57	19.06	1830	6.78%	5.22%
CIC	8	0:01:27	0:11:41	9.38	75	0.56%	0.43%
Organisational consultation directly	129	0:00:34	1:13:59	19.24	2482	9.02%	2.70%
Organisational consultation Telephone	30	0:00:42	0:21:21	7.87	236	2.10%	0.78%
Patient safety check	14	0:00:53	0:12:22	4.86	68	0.98%	0.45%
Teaching	53	0:01:37	1:26:07	16.62	881	3.71%	3.15%
Handover	2	0:02:42	0:05:25	42.50	85	0.14%	0.20%
Others	*7*	*0:02:16*	*0:15:53*	*50.71*	*355*	*0.50%*	*0.60%*
Blood gas analysis	6	0:01:46	0:10:39	59.17	355	0.42%	0.39%
Others	1	0:05:14	0:05:14	0.00	0	0.07%	0.19%
Follow-up	*207*	*0:01:04*	*3:41:42*	*15.99*	*3262*	*14.50%*	*8.10%*
Clean up/disposal/ Contact surface disinfection	150	0:01:15	3:07:33	10.43	1565	10.49%	6.85%
Disinfection and reconditioning	6	0:00:45	0:04:32	8.00	48	0.42%	0.17%
Return material/equipment	51	0:00:34	0:29:36	32.33	1649	3.57%	1.08%
Organising	*160*	*0:01:39*	*4:26:14*	*19.31*	*3051*	*11.20%*	*9.70%*
Refill routine	49	0:03:03	2:29:53	25.00	1225	3.43%	5.48%
Hygiene routine	17	0:01:24	0:23:57	7.53	128	1.19%	0.88%
Information procurement	94	0:00:58	1:32:25	18.06	1698	6.57%	3.38%
Patient care	*53*	*0:02:34*	*2:16:49*	*5.50*	*286*	*3.70%*	*5.00%*

	n	Ø Mean Duration	Σ Total Duration	Ø Mean Steps	Σ Total Steps	Proportion of Total Count	Proportion of Total Duration
Break	*14*	*0:06:40*	*1:33:33*	*27.21*	*381*	*1.00%*	*3.40%*
Preperation	*301*	*0:01:48*	*9:06:44*	*19.86*	*5840*	*21.00%*	*20.00%*
Get material/equipment	154	0:00:58	2:30:39	29.21	4499	10.77%	5.51%
Prepare material/equipment	147	0:02:41	6:36:05	9.12	1341	10.28%	14.48%
**Total**	**1430**	**0:01:54**	**45:36:13**	**16.81**	**23326**	**100.00%**	**100.00%**

Table S2 Secondary activities

	n	Ø Mean Duration	Σ Total Duration	Ø Mean Steps	Σ Total Steps	Proportion of Total Count	Proportion of Total Duration
Assistance	*5*	*0:01:32*	*0:07:44*	*1.00*	*5*	*1.70%*	*2.90%*
Emergence	2	0:02:15	0:04:31	2.50	5	0.70%	1.70%
Induction	3	0:01:04	0:03:13	0.00	0	1.00%	1.20%
Documentation	*14*	*0:00:47*	*0:11:09*	*0.00*	*0*	*4.80%*	*4.20%*
Patientrecord	12	0:00:37	0:07:26	0.00	0	4.10%	2.80%
QM	2	0:01:51	0:03:43	0.00	0	0.70%	1.40%
Communication	*216*	*0:00:54*	*3:17:32*	*1.32*	*283*	*74.00%*	*74.50%*
Work dialogue	39	0:01:12	0:47:04	0.00	0	13.36%	17.76%
CIC	7	0:01:03	0:07:22	0.00	0	2.40%	2.80%
Organisational consultation directly	52	0:00:30	0:26:48	0.50	26	17.80%	10.10%
Organisational consultation Telephone	57	0:00:32	0:30:27	0.63	36	19.50%	11.50%
Patient safety check	5	0:00:33	0:02:45	0.00	0	1.70%	1.00%
Teaching	56	0:01:29	1:23:05	3.95	221	19.20%	31.30%
Follow-up	*9*	*0:00:37*	*0:05:39*	*0.00*	*0*	*3.10%*	*2.10%*
Clean up/disposal/ Contact surface disinfection	7	0:00:42	0:04:59	0.00	0	2.40%	1.90%
Return material/equipment	2	0:00:20	0:00:40	0.00	0	0.70%	0.30%
Organising	*18*	*0:00:52*	*0:15:36*	*2.00*	*36*	*6.20%*	*5.90%*
Refill routine	2	0:01:25	0:02:51	0.00	0	0.70%	1.10%
Hygiene routine	3	0:00:43	0:02:11	0.00	0	1.00%	0.80%

	n	Ø Mean Duration	Σ Total Duration	Ø Mean Steps	Σ Total Steps	Proportion of Total Count	Proportion of Total Duration
Information procurement	13	0:00:48	0:10:35	2.77	36	4.50%	4.00%
Patient Care	*10*	*0:00:40*	*0:06:40*	*2.50*	*25*	*3.40%*	*2.50%*
Preperation	*20*	*0:01:02*	*0:20:40*	*0.00*	*0*	*6.80%*	*7.80%*
Get material/equipment	1	0:00:43	0:00:43	0.00	0	0.30%	0.30%
Prepare material/equipment	19	0:01:03	0:19:57	0.00	0	6.50%	7.50%
**Total**	**292**	**0:00:54**	**4:25:01**	**1.20**	**349**	**100.00%**	**100.00%**

Table S3 Interruptions

	n	Ø Mean Duration	Σ Total Duration	Ø Mean Steps	Σ Total Steps	Proportion of Total Count	Proportion of Total Duration
Communication	*126*	*0:00:25*	*0:53:53*	*2.18*	*275*	*71.19%*	*50.41%*
Work dialogue	7	0:00:37	0:04:21	0	0	3.95%	4.07%
CIC	2	0:00:28	0:00:57	0	0	1.13%	0.89%
Organisational consultation directly	34	0:00:24	0:13:44	7.06	240	19.21%	12.84%
Organisational consultation Telephone	83	0:00:25	0:34:52	0.42	35	46.89%	32.61%
Material	*19*	*0:00:56*	*0:17:44*	*21.42*	*407*	*10.73%*	*16.59%*
missing	19	0:00:56	0:17:44	21.42	407	10.73%	16.59%
Emergency	*1*	*0:00:47*	*0:00:47*	*5*	*5*	*0.56%*	*0.73%*
Technology	*6*	*0:00:52*	*0:05:14*	*0.83*	*5*	*3.39%*	*4.89%*
Alarm monitoring	1	0:00:21	0:00:21	0	0	0.56%	0.33%
Technology malfunction	5	0:00:58	0:04:53	1	5	2.82%	4.57%
Accidental	*9*	*0:00:31*	*0:04:44*	*0*	*0*	*5.08%*	*4.43%*
Delays	*16*	*0:01:32*	*0:24:32*	*0*	*0*	*9.04%*	*22.95%*
Information missing	5	0:00:55	0:04:35	0	0	2.82%	4.29%
Waiting	11	0:01:48	0:19:57	0	0	6.21%	18.66%
**Total**	**177**	**0:00:36**	**1:46:55**	**3.91**	**692**	**100.00%**	**100.00%**

Table S4 Descriptive statistics clustered by observation session

	Min	Max	Mean	SD	99%-CI lower limit	99%-CI upper limit
Interruptions n/h	0.00	9.94	3.93	0.55	2.42	5.43
Interruptions min/h	0.00	5.40	2.31	0.34	1.36	3.26
Secondary activities n/h	0.58	18.52	6.14	0.87	3.74	8.54
Secondary activities min/h	0.25	28.64	5.52	1.05	2.62	8.43
Steps/h	76.94	1038.83	517.90	41.86	402.53	633.28

Since individual observation sessions varied slightly in duration, data are reported as x/h.

Table S5 Descriptive statistics for the questionnaire

	N	Min	Max	Mean	SD
Before Observation Personnel Situation	30	0	8	2.00	2.259
Before Observation Fatigue	30	0	10	3.70	3.175
Before Observation Staffing Levels	30	0	10	5.97	3.068
After Observation Job Satisfaction	30	1	10	8.07	2.434
After Observation Error Potential Due to Interruptions	30	0	9	2.93	2.815
After Observation Subjective Stress Level	30	0	8	2.30	2.628

Note: Scale: Not at all = 0; Absolutely = 10

Table S6 Spearman's rank correlation coefficients between workflow characteristics and self-reported measures at the observation session level (N = 30)

Variable	1	2	3	4	5	6	7	8	9	10	11	12	13
**Workflow and contextual characteristics**													
1. Work experience (yrs)	1												
2. Shift management (y/n)	.45	1											
3. Non-occupational stress	-.46	-.51	1										
4. Tiredness	-.19	-.16	**.64****	1									
5. Personnel Situation	-.03	.22	-.20	-.30	1								
**Self-reported outcomes**													
6. Job satisfaction	.36	.53	**-.62***	**-.63***	.3	1							
7. Error potential	-.29	-.21	.47	.43	-.27	-.58	1						
8. Work-related stress level	-.39	-.36	**.65****	49	-.33	-.52	**.74****	1					
**Observed workflow measures**													
9. Interruptions n/h	.30	.35	-.24	-.32	.08	.16	.08	-.19	1				
10. Interruptions min/h	-17	.17	-.08	-.21	.02	-.02	.13	-.06	**.88****	1			
11. Secondary activities n/h	-22	-29	-.02	.09	.09	.18	-.02	-.10	.18	.04	1		
12. Secondary activities min/h	.20	.11	.09	.04	-.09	.06	-.03	-.18	.22	.08	**.84****	1	
13. Steps walked	.34	.22	-.27	-.24	-.26	.17	-.15	-.25	.29	.20	-.05	.21	1

* p < .000347222 (after Bonferroni–Holm adjustment of alpha = .05), ** p < 0.00006757 (for adjusted alpha = .01)
